# Risk factors assessment for nasal colonization of *Staphylococcus aureus* and its methicillin resistant strains among pre-clinical medical students of Nepal

**DOI:** 10.1186/s13104-016-2021-7

**Published:** 2016-04-12

**Authors:** Shamshul Ansari, Rajendra Gautam, Sony Shrestha, Safiur Rahman Ansari, Shankar Nanda Subedi, Muni Raj Chhetri

**Affiliations:** Department of Microbiology, Chitwan Medical College, Bharatpur, Chitwan, Nepal; Asian College for Advanced Studies, Satdobato, Kathmandu, Nepal; Department of Community Medicine and Public Health, Chitwan Medical College, Bharatpur, Chitwan, Nepal

**Keywords:** Medical students, MRSA, Nasal colonization, Risk factors, *Staphylococcus aureus*

## Abstract

**Background:**

*Staphylococcus aureus* (*S. aureus*), a normal flora of nasal cavity, can cause minor to life threatening invasive diseases and nosocomial infections. Methicillin resistant strains of *S. aureus* are causing a great challenge for treatment options. Therefore, the purpose of this study was to assess the nasal carriage rate of *S. aureus*, its methicillin resistant strains and risk factors in medical students prior to clinical exposure.

**Methods:**

The bacterial growth of *S. aureus* from nasal swab culture was identified by using standard microbiological methods recommended by American Society for Microbiology. Modified Kirby–Bauer disk diffusion method was used for antibiotic susceptibility testing and methicillin resistance was confirmed using cefoxitin and oxacillin disks. D-zone test method was used to determine the inducible clindamycin resistance.

**Results:**

Among 200 participants, nasal carriage of *S. aureus* was detected from 30 (15 %) subjects. Upper respiratory tract infections significantly (P < 0.05) contributed the carriage of *S. aureus* and their methicillin resistant strains. All of the isolates were reported to be susceptible to vancomycin and teicoplanin. *S. aureus* strains detected from 8 (4 %) students were confirmed to be methicillin resistant.

**Conclusions:**

The result of our study demands for strict policy to screen all the students for nasal carriage of *S. aureus* and its MRSA strains to minimize the transmission of this organism from community to hospital settings.

## Background

*Staphylococcus aureus* (*S. aureus*) is a normal flora of moist squamous epithelium of the anterior nares. Majority of the populations (60 %) are intermittent carriers while 20 % of the population is always colonized with *S. aureus* and 20 % of populations never carry this organism [1]. The evidence suggests that the populations harboring *S. aureus* and its methicillin resistant (MRSA) strains are at higher risk for developing invasive infection [2–4].

A range of minor as well as life threatening conditions like skin infections (pimples, impetigo, boils, cellulitis, folliculitis, carbuncles, scalded skin syndrome, abscesses), pneumonia, meningitis, osteomyelitis, endocarditis, toxic shock syndrome (TSS), and septicemia can be caused by *S. aureus* [5]. Coagulase negative *Staphylococcus* species (CoNS) act as the most common causative agents of nosocomial bacteremia [6].

Nasal carriage of MRSA contributes as a major risk factor for subsequent infection and transmission of this pathogen [7, 8]. Prolonged hospitalization, antibiotics exposure, and the presence of other patients with MRSA colonization or infection in the hospital are the major risk factors for acquiring MRSA infections. MRSA causes life threatening infections and greater mortality than that from methicillin-sensitive *S. aureus* (MSSA) infections. Vancomycin, a glycopeptide antibiotic is the drug of choice to treat MRSA infections. However, the continuous use of vancomycin may promote growth of vancomycin-intermediate *S. aureus* (VISA) and vancomycin-resistant *S. aureus* (VRSA) [9]. Therefore, this study was conducted to assess the nasal carriage rate of *S. aureus*, MRSA, its antimicrobial susceptibility profile and the associated risk factors in the medical students prior to their clinical exposure.

## Methods

This simple and cross-sectional study was carried out at Department of Microbiology, Chitwan Medical College (having a 600 bed teaching hospital) in the city of Bharatpur, Chitwan District, Narayani Zone, Nepal in March, 2014. A total 200 medical students who were studying in their first year of their medical education and were not exposed to their clinical posting were enrolled in this study.

### Data collection

After obtaining the appropriate written consent the participants were requested to respond to a questionnaire on basic *demographic characteristics* (gender, residence, number of family members, profession of family, family income, family education), *any potential risk factors* (history of hypertension, renal disease, lower respiratory tract infection (LRTI), gastro-intestinal (GI) disease, upper respiratory tract infections (URTIs), recent surgery, recent hospitalization, recent visit to out-patient departments (OPD), any recent medication, recent antibiotic use, recent visit to hospital admitted family members) and *habitual factors* (vehicle used by participants, vehicle used by participants’ family members, recent visit to public amusement places, tattoo or acupuncture, alcohol consumption habit, contact with livestock and pets) during swab collection.

### Exclusion criteria

The participants receiving either intranasal antibiotic ointment including mupirocin, or antistaphylococcal antibiotics including clindamycin, cephalexin, cefazolin, oxacillin, dicloxacillin, trimethoprim/sulfamethoxazole, linezolid or vancomycin within 2 weeks were excluded in this study.

### Sample collection

Nasal swabs were collected from both anterior nares of the students by using moist cotton swabs. Aseptic technique (disinfecting outer nostrils with alcohol) was followed while collecting the swab samples. The swab samples were transported immediately to the laboratory and processed for bacteriological profile within half an hour of collection.

## Microbiological study

### Swab processing

For the isolation of *S. aureus*, the collected swab samples were gently rolled and streaked on the 5 % sheep blood agar (BA), DNase agar and mannitol salt agar (MSA) plates (HiMedia Laboratories Pvt. Limited, India). The inoculated BA, DNase and MSA plates were incubated at 37 °C for up to 24 h as described in previously published article [10]. Identification of *S. aureus* was carried out following standard microbiological methods recommended by American Society for Microbiology (ASM) [11].

### Antibiotic susceptibility testing

Modified Kirby–Bauer disk diffusion method was used for antibiotic susceptibility testing and isolates were considered either sensitive or resistant in compliance with Clinical and Laboratory Standards Institute (CLSI) guidelines [12]. Several antibiotics (HiMedia Laboratories, Pvt. Limited, India) used were: penicillin G (10 U), ciprofloxacin (5 μg), gentamicin (10 μg), amikacin (30 μg), erythromycin (15 μg), co-trimoxazole (25 μg), tetracycline (10 μg), rifampicin (15 μg), vancomycin (30 μg), teicoplanin (30 μg), cefoxitin (30 μg), oxacillin (1 μg) and clindamycin (2 μg).

### Identification of methicillin resistant strains (MRSA)

Disk approximation method was carried out by using oxacillin (1 μg) and cefoxitin (30 μg) disks for identification of methicillin resistance in *S. aureus*. The strains showing the diameter of the zone of inhibition (ZOI) of ≤10 mm with the oxacillin disk or ≤21 mm with the cefoxitin disk were recorded as methicillin resistant as recommended by CLSI [13].

### Identification of inducible clindamycin resistance in *S. aureus*

Identification of inducible macrolide–lincosamide–streptogramin B (iMLSB) resistance in *S. aureus* was also performed by disk approximation method. The method involved inoculation of *S. aureus* isolate on Mueller–Hinton agar plate and placing clindamycin (CLI) and erythromycin (ERY) disks approximately 15 mm apart (measured edge to edge); the plate was then incubated for 16–18 h. The zone of inhibition around the CLI disk proximal to the ERY disk (producing a zone of inhibition shaped like the letter D) showing flattening was considered a positive result and indicates the inducible CLI resistance by ERY (a positive “D-zone test”) [13].

*S. aureus* ATCC 25923 (for antibiotic sensitivity and negative control of methicillin resistance) and ATCC 43300 (for positive control of methicillin resistance) were used as a control organism [10].

### Statistical analysis

Statistical analysis was performed using SPSS-16 version. Differences in proportions were assessed by Chi square test. P values less than 0.05 were considered statistically significant.

## Results

### Participants and positive rates

The participated population in this study included 200 (male 105 and female 95) students with male to female ratio of 1.1. *S. aureus* strains were isolated from 30 participants giving a prevalence rate of 15 % and out of 30 *S. aureus* strains 8 were identified as MRSA strains giving the prevalence rate of 4 %.

### Participants with socio-demographic risk factors

Although the colonization rate in rural residents is higher (20.5 %) than urban residents (13.7 %), the association was not statistically significant. Non-significantly higher colonization rate was also found in participants having more than 4 family members (Table 1).

**Table 1:** Participants with socio-demographic characteristics

Variables	Total participants (%)	Positive participants (%)	P values
Gender			
Male	105 (52.5)	14 (13.3)	0.488
Female	95 (47.5)	16 (16.8)	
Residence			
Rural	39 (19.5)	8 (20.5)	0.283
Urban	161 (80.5)	22 (13.7)	
No. of family members			
More than 4	106 (53.0)	17 (16.0)	0.660
Up to 4	94 (47.0)	13 (13.8)	
Profession of family			
Health care	39 (19.5)	4 (10.3)	0.458
Others	161 (80.5)	26 (16.1)	
Family income			
Low	15 (7.5)	1 (6.7)	
Middle	154 (77.0)	25 (16.2)	
High	31 (15.5)	4 (12.9)	
Family education			
Up to high school	32 (16.0)	7 (21.9)	0.237
College	168 (84.0)	23 (13.7)	

### Participants and potential risk factors

Although all of the participants with LRTIs and renal disease were found colonized with *S. aureus* due to its small sample size (n = 1), the association was not statistically significant while 25 % of participants having URTIs were significantly colonized with this organism (P = 0.035) (Table 2).

**Table 2:** Participants with potential risk factors

Variables	Total participants (%)	Positive participants (%)	P values
Hypertension			
Yes	4 (2.0)	1 (25.0)	0.480
No	196 (98.0)	29 (14.8)	
Renal disease			
Yes	1 (0.5)	1 (100.0)	0.15
No	199 (99.5)	29 (14.6)	
LRTI			
Yes	1 (0.5)	1 (100.0)	0.15
No	199 (99.5)	29 (14.6)	
Gastro-intestinal disease			
Yes	17 (8.5)	1 (5.9)	0.477
No	183 (91.5)	29 (15.8)	
URTI			
Yes	44 (22.0)	11 (25.0)	*0.035*
No	156 (78.0)	19 (12.2)	
Recent surgical procedures			
Yes	2 (1.0)	0 (0)	
No	198 (99.0)	30 (15.2)	
Recent admission to hospital			
Yes	8 (4.0)	0 (0)	
No	192 (96.0)	30 (15.6)	
Recent visit to out-patient departments			
Yes	50 (25.0)	4 (8.0)	0.168
No	150 (75.0)	26 (17.3)	
Recent medication			
Yes	42 (21.0)	7 (16.7)	0.734
No	158 (79.0)	23 (14.6)	
Recent antibiotic use			
Yes	51 (25.5)	8 (15.7)	0.874
No	149 (74.5)	22 (14.8)	
Recent visit to hospital admitted family members			
Yes	29 (14.5)	6 (20.6)	0.354
No	171 (85.5)	24 (14.0)	

Recent—in last 3 months

### Participants and habitual risk factors

Habitual risk factors like vehicle used by participants and their family members, recent visit to public amusement places, alcohol consumption, contact with livestock and pet did not contribute for the colonization of *S. aureus* (Table 3).

**Table 3:** Participants with habitual risk factors

Variables	Total participants (%)	Positive participants (%)	P values
Vehicle used by participants			
Public	168 (84.0)	25 (14.9)	0.920
Personal	32 (16.0)	5 (15.6)	
Vehicle used by participant’s family members			
Public	82 (41.0)	12 (14.6)	0.904
Personal	118 (59.0)	18 (15.3)	
Recent visit to public amusement places			
Yes	106 (53.0)	12 (11.3)	0.122
No	94 (47.0)	18 (19.1)	
Tattoo or acupuncture			
Yes	0 (0)	0 (0)	
No	200 (100)	30 (15.0)	
Alcohol consumption habit			
Yes	5 (2.5)	1 (20.0)	0.560
No	195 (97.5)	29 (14.9)	
Contact with livestock			
Yes	26 (13.0)	2 (7.7)	0.263
No	174 (87.0)	28 (16.0)	
Contact with pets			
Yes	60 (30.0)	9 (15.0)	1.0
No	140 (70.0)	21 (15.0)	

### Antimicrobial susceptibility testing profile

Table 4 shows the antimicrobial resistance profile of 30 *S. aureus* strains isolated in this study. Among 30 tested isolates, resistance to penicillin G was most common (73.0 %) and frequent resistance was also noted with ciprofloxacin (36.7 %). Eight (26.7 %) isolates were resistant to methicillin by cefoxitin and oxacillin disk method and 4 (13.3 %) isolates were found to have inducible clindamycin resistance by D-zone test. No isolates were found resistant to vancomycin and teicoplanin in this study. As expected, all of the MRSA strains were resistant to penicillin G (Table 4).

**Table 4:** Antibiotic resistance pattern of *S. aureus* and MRSA

Antibiotics	*S. aureus* (n = 30) Resistant frequency (%)	MRSA (n = 8) Resistant frequency (%)
Penicillin G	22 (73)	8 (100)
Ciprofloxacin	11 (36.7)	6 (75.0)
Gentamicin	10 (33.3)	5 (62.5)
Amikacin	3 (10)	1 (12.5)
Erythromycin	10 (33.3)	5 (62.5)
Cotrimoxazole	6 (20)	4 (50.0)
Tetracycline	6 (20)	3 (37.5)
Rifampicin	6 (20)	3 (37.5)
Vancomycin	0	0
Teicoplanin	0	0
Cefoxitin	8 (26.7)	8 (100)
Oxacillin	8 (26.7)	8 (100)
Clindamycin	8 (26.7)	3 (37.5)

### MRSA and associated risk factors

MRSA strains were detected from 8 (4 %) participants. Males were more likely to carry MRSA (62.5 %) than females but association was not significant (P = 0.295). However, URTIs, recent visit to public amusement places, contact with pet (dog) and rural type of residence were listed as significant contributing risk factors for MRSA colonization (Table 5).

**Table 5:** Risk factors and MRSA positive cases (N = 8)

Variables	Positive numbers (n = 8)	P value
Gender		
Male	5	0.295
Female	3	
Co-morbidities		
URTIs	6	*0.009*
Others	2	
Recent visit to hospital admitted family members		
Yes	1	0.536
No	7	
Recent visit to public amusement places		
Yes	6	*0.018*
No	2	
Contact with pet		
Yes (dog)	6	*0.0005*
No	2	
Type of residence		
Urban	3	*0.007*
Rural	5	
Vehicle used by participant’s family member		
Personal	5	0.866
Public	3	
Profession of family		
Health care	2	0.257
Other than health care	6	
Family income		
Middle	8	0.140
Others	0	

## Discussions

Now-a-days, the rate of urbanization is increasing in search of better life style, jobs, medical facilities and better education facilities. The result of our study also indicates that majority of the participants (80.5 %) belonged to urban area and 19.5 % of the participants belonged to family having health care professions. As pets are being taken as close friends to humans, 60 (30 %) participants of our study were having pets (mostly dog). Common cold, a minor form of URTIs, is common in Nepal and thus, 44 (22 %) of the participants were suffering from upper respiratory tract infections (URTI) during the study period.

Although, large proportion of *S. aureus* carriage is through the anterior nares of the nasal passages, it can be found on the skin of the host [1]. The combination of defective host immunity and the bacterial ability to evade host innate immunity results in the ability of the nasal passages to harbor *S. aureus* [14]. Approximately 20 % of individuals act as persistent carriers and almost always carry one type of strain [15]. In this study, we detected 15 % nasal carriage of *S. aureus* in medical students, similar to the rates detected from Malaysia [16, 17] and Iraq [18].

In this study, a non-significant association was observed between the *S. aureus* colonization and type of residence. The present analysis found that 73.3 % of the students positive for nasal colonization of *S. aureus* were from cities and 83.3 % of participants used public vehicle for travelling. The changing pattern of life style in urban area like frequent visit of shopping malls and theatres, attending parties and travelling via public vehicles bring peoples to be in close contact and easily can transmit the pathogens to others. Moreover, contaminated door handles of public vehicles also act as source infections.

Although, non-significant association was observed between nasal carriage of *S. aureus* and clinical factors such as medication and antibiotic use in last 3 months, there was significant association (P = 0.035) with upper respiratory tract (nasal) infections in our study. Studies from other settings have also reported increased spread of *S. aureus* during an episode of URTIs [19]. In a study conducted from Malaysia, 22.5 % cases of nasal carriage were associated with URTIs and 9.9 % cases were associated with recent antibiotic use [17].

The reports of recent study have documented that *S. aureus* can survive on dogs and cats [20, 21]. Some authors believe that health-care workers’ dogs should be considered a significant source of antibiotic-resistant *S. aureus*, especially during outbreaks [20]. In our study, we found that 30 % of the nasal carriers of *S. aureus* have had contact with pet (mostly dog), the findings being much lower than the result of 77 % from Virginia [22]. The lower rate of our study may be due to the rather still uncommon practice of domesticating pets in Nepal than others.

Although, the overall resistance rates to commonly prescribed antibiotics in isolates were below 50 %, as expected the rate was high (73 %) with penicillin because only a small proportion of the *S. aureus* lineages do not produce beta-lactamases [23–26]. Similarly, a higher rate of resistance (92 %) to ampicillin was also reported in a study conducted in Brazil [27].

Ciprofloxacin became the most widely used quinolone antibiotic after its introduction into clinical use in the late 1980s and early 1990s [28, 29]. In recent years, resistance has been developed in many bacteria, making it significantly less effective [30, 31]. We identified ciprofloxacin resistance rate of 36.7 % in this study much higher than the result (8.8 %) from Brazil [27]. This high rate of resistance in our study may be because of its indiscriminate use in our setting as a consequence of low cost and easy availability. Once effective against staphylococcal infections, the aminoglycoside antibiotics such as kanamycin, gentamicin, streptomycin etc., have been found less effective because of the development of mechanisms to inhibit the action, which occurs via protonated amine and/or hydroxyl interactions with the ribosomal RNA of the bacterial 30S ribosomal subunit [32]. As a consequence of low cost and easy availability, there has been indiscriminate use of this antibiotic similarly as with ciprofloxacin in our context. We observed the rate of gentamicin resistance as 33.3 % and amikacin resistance as 10 %. Similar rate of gentamicin resistance (25 %) was also detected by Sharma et al. [33] whereas lower rate of amikacin resistance (4 %) was also reported from Brazil [27].

Today, the therapeutic roles of erythromycin and trimethoprim–sulfamethoxazole (co-trimoxazole) are increasingly limited due to its extensive use for the treatment of both minor and serious staphylococcal infections. One third (33.3 %) of our isolates were resistant to erythromycin and 20 % isolates were resistant to co-trimoxazole, the results are in agreement with the reported finding from Iran [34].

Glycopeptides like vancomycin and teicoplanin could be reserved for the management of MRSA infections because of its high efficacy in virtually all isolates of *S. aureus* [35, 36]. The result of susceptibility in the current study to vancomycin and teicoplanin is comparable to that of other studies conducted worldwide [18, 27, 33]. The promising efficacy of glycopeptides is probably due to high cost and low usage of these regimens in Nepal. However, increased resistance to teicoplanin has been reported overseas [37–40]. Thus, glycopeptides especially vancomycin can be used empirically for serious staphylococcal infections while waiting for susceptibility testing results to come through [41].

Methicillin, the first antibiotic of β-lactamase-resistant penicillins (methicillin, oxacillin, cloxacillin, and flucloxacillin) was detected to be ineffective only after two years of its introduction (introduced in 1959) in England and it was the first case of MRSA [42]. Then until 1990s, when there was an explosion in MRSA prevalence in hospitals, MRSA generally remained an uncommon finding, [43]. In present study, two different methods, the cefoxitin disk and oxacillin disk methods were employed for the detection of MRSA. According to CLSI guidelines, the *mecA* mediated resistance to oxacillin can be detected by using cefoxitin disk or oxacillin disk method but cefoxitin disk method is more preferred because it is easier to read and also cefoxitin acts as an inducer of the *mecA* gene [13]. Results of the present study indicated that, 4 % (8/200) students harbored the MRSA in their nasal cavity. Similarly, 6 % MRSA carriage rate in medical students before clinical exposure was also detected from India [44].

Macrolide-resistant isolates of *S. aureus* may be resistant to only macrolides mediated by efflux mechanism encoded by the *msrA* gene or may have constitutive or inducible resistance to clindamycin mediated by methylation of the 23S rRNA encoded by the *erm* gene [13]. Clindamycin is considered as one of the drugs of choice in *S. aureus* infections but an *erm* gene mediated inducible resistance may result in treatment failure [45]. In our study, we identified 4 isolates (13.3 %) as D-zone test positive, indicating inducible resistance to clindamycin. Similar result of inducible resistance to clindamycin was also reported from Nepal [10].

Spread of *S. aureus* (including MRSA) is generally through human-to-human contact, although recently some veterinarians have documented that the infection can also spread through pets [46]. In a case control study, 73 % of MRSA were recovered from pets (cat, dog and other rodents) [22]. Similarly, in our study we observed that 75 % of MRSA carriers have had contact with pet especially dog. High proportion of MRSA (75 %) colonization was also significantly associated with URTIs (P = 0.009) and visit to public amusement places (P = 0.018). Although the resident type was not associated with the colonization rate of *S. aureus*, it did the colonization of MRSA in our study (P = 0.007).

Our study had a few limitations, we selected a limited pool of antibiotics for susceptibility testing and molecular studies could not performed to confirm MRSA isolates due to financial constraints.

## Conclusions

The result of this study highlights the nasal carriage of *S. aureus* and their methicillin resistant counterparts in the medical students. This study indicates that carriage of this organism has no significant association with socio-demographic and habitual risk factors. However, URTIs can enhance the carriage of *S. aureus* as well as their MRSA strains. Recent visit to public amusement places, contact with dog (pet) and rural residence were also documented as the significant risk factors contributing the MRSA colonization. The colonization of *S. aureus* and MRSA can play a key role in the epidemiology and pathogenicity of community as well as hospital associated infections. From this study, the longitudinal surveillance of nasal carriage of *S. aureus* should be made an essential protocol to minimize the transmission of this organism from community to hospital and vice versa.

## Abbreviations

MRSA: methicillin resistant *Staphylococcus aureus*
MSSA: methicillin susceptible *Staphylococcus aureus*
VRSA: vancomycin resistant *Staphylococcus aureus*
VISA: vancomycin intermediate *Staphylococcus aureus*
ATCC: American type culture collection
OPD: outpatient department
URTIs: upper respiratory tract infections
LRTIs: lower respiratory tract infections
GI: gastrointestinal
ASM: American Society for Microbiology
TSS: toxic shock syndrome
CLSI: Clinical and Laboratory Standard Institute
BA: blood agar
MSA: mannitol salt agar
ZOI: zone of inhibition
IRC: Institutional Review Committee
